# Investigating the Influence of Experimentally Induced Secondary Hyperalgesia on Pain Prediction Error Encoding in Healthy Individuals: A Novel Virtual Reality Protocol

**DOI:** 10.1002/ejp.70113

**Published:** 2025-08-22

**Authors:** Federico Palmisani, Jawahar Sri Prakash Thiyagarajan, Joe Horsey, Sam W. Hughes, Sonia Medina

**Affiliations:** ^1^ Department of Clinical and Biomedical Sciences University of Exeter Exeter UK

## Abstract

**Background:**

Persistent mismatches between predicted and actual pain‐related signals, namely prediction errors (PEs), can cause maladaptive overestimation of pain intensity, a common feature of chronic pain states. Experimental protocols used to assess the contribution of central sensitisation (CS) to dysregulated prediction systems are lacking. To address this, we implemented a novel virtual reality (VR) paradigm to evoke PEs during mechanical stimulation following experimentally induced CS via the high‐frequency stimulation (HFS) model.

**Methods:**

Twenty healthy volunteers underwent HFS on the right forearm. Mechanical pain sensitivity (MPS) was assessed through pinprick stimuli before and 30 min post‐HFS to evaluate secondary hyperalgesia. Following this, participants received mechanical stimuli at proximal (sensitised area) and distal (non‐sensitised area) points from the HFS site, with visual cues presented on their arm via VR alongside hand tracking technology indicating the stimulus location, allowing participants to make pain predictions. Cues were either congruent (matching) or incongruent (mismatching) with the actual stimulus site to evoke PEs.

**Results:**

Results showed that MPS significantly increased following HFS, confirming secondary hyperalgesia. Stimuli in sensitised areas induced more pain than in non‐sensitised areas. Incongruent cues successfully elicited PEs across all locations; however, expectations modulated pain perception only in non‐sensitised areas. Similarly, during incongruent trials, PEs diminished over time (reflecting adaptive learning) only in non‐sensitised areas.

**Conclusions:**

These data demonstrate that pain expectations can influence pain perception differently in centrally sensitised and non‐sensitised states. We propose this protocol as a good candidate to assess how cognitive and psychological manipulations influence PEs at various stages of CS.

**Significance Statement:**

We introduce a novel VR paradigm to show that secondary hyperalgesia alters how pain expectations and prediction errors influence pain perception, highlighting distinct adaptive learning patterns.

## 1 Introduction

The predictive coding model of pain suggests that the brain continuously anticipates sensory input to prepare for adaptive responses (Song et al. 2019; Tabor and Burr 2019). Predictions are shaped by prior learning and expectations (Chen 2023; Lersch et al. 2023). When the predicted pain intensity mismatches pain perception in the presence of a given sensory input, prediction errors (PEs) arise (Chen 2023).

PEs allow one to adjust future predictions, dynamically modulating incoming signals to fine‐tune pain responses based on whether it has underestimated or overestimated the incoming threat, aiding protective reflexes (Tabor and Burr 2019). However, during chronic pain, individuals may begin to overestimate the danger posed by sensory stimuli due to fear of pain (Vlaeyen 2015) and pain catastrophising (Haythornthwaite et al. 2024; Yakunchikov et al. 2016). On the other hand, persistent afferent signal amplification at the dorsal horn, known as central sensitisation (CS), can lead to heightened sensitivity to both noxious and non‐noxious mechanical stimuli (Baron et al. 2013; Latremoliere and Woolf 2009), further biasing the sense of immediate threat (Hollander et al. 2010; Timmers et al. 2019) and making the brain overly sensitive to pain, even in the absence of nociceptive input.

Understanding the specific role of CS in driving persistent PEs, where the brain fails to adjust its pain predictions despite changes in sensory input, is therefore crucial for designing personalised treatments (Knaggs 2016) that target the underlying root of maladaptive pain learning (Bannister and Hughes 2023; Mansour et al. 2014). Nevertheless, experimental protocols able to assess this are currently lacking. The high‐frequency stimulation (HFS) model can induce prolonged secondary hyperalgesia in healthy individuals within a well‐defined heterotopic area (Fawsitt‐Jones et al. 2024; Meijs et al. 2025; van den Broeke and Mouraux 2014), a phenomenon attributed to CS. Considering this, we introduce a novel approach where we can induce PEs following HFS by presenting visual cues that indicate the location of upcoming stimuli, either inside or outside of the sensitised area, and manipulating the congruency between the cue and the actual stimulation site that gives rise to PEs. By integrating virtual reality (VR) with hand‐tracking technology (Clark 2022), we can deliver visual cues with a high level of experimental control (Medina et al. 2024) while inducing high levels of embodiment (Salagean et al. 2022), as it allows participants to continuously view a virtual representation of their arm while remaining unaware of the actual location of the stimuli in relation to the cued site. We propose that this strategy can allow us to test how the discrepancy between predicted and perceived pain (i.e., PEs) interacts with the presence of secondary hyperalgesia to result in different levels of perceived pain.

In this study, we explored pain modulatory responses due to experimentally induced PEs following HFS using VR in a healthy cohort. Our preregistered hypotheses (Palmisani et al. 2024) were: (i) HFS would increase mechanical pain sensitivity (MPS) measures; (ii) PEs would influence pain perception: if predicted pain at the attended location is lower than perceived pain, the result would be lower pain perception compared to a match between predicted and perceived pain. If predicted pain is higher than perceived pain, the result would be higher pain perception compared to a match between predicted and perceived pain. Crucially, we set out to explore how these bidirectional relationships vary depending on whether stimulation is delivered in a sensitised versus non‐sensitised area.

## 2 Methods

All methods and data analyses adhered to the preregistered study plan available on OSF (see Palmisani et al. 2024).

### 2.1 Participants

A total of 20 healthy individuals, aged between 20 and 31 (mean age: 25, SD = 2, 15 males), participated in this study. The sample included individuals aged over 18 years identifying as healthy. Exclusion criteria included the presence of chronic illnesses or current pain, a history of epilepsy, substance or alcohol abuse, skin conditions such as eczema, and any ongoing psychological conditions requiring psychoactive medications, unless the dosage had remained stable for at least 3 months. Additionally, participants were excluded if they consumed more than eight caffeinated beverages or smoked more than five cigarettes per day. Participants were instructed to abstain from alcohol, nicotine, painkillers, and all but a single caffeinated drink on the day of the study. Each participant provided written informed consent prior to participating in the study. The experiment was approved by the Health Research Authority and Health and Care Research Wales Ethics Committee (reference: 22/HRA/4672).

### 2.2 Experimental Design

This study employed a repeated‐measures, within‐subject design to investigate the influence of top‐down predictive processes on pain perception during peripherally induced central sensitisation. Each participant completed a single session in the laboratory. Compliance with study lifestyle guidelines was assessed at the beginning of the session. In order to ensure the absence of any ongoing potentially confounding pain, participants also answered the question ‘Are you in any pain today’ with a numerical score on a numerical rating scale (NRS) that ranged from 0 (‘no pain at all’) to 100 (‘worst pain imaginable’). All participants underwent study procedures in the same order.

### 2.3 HFS Model

Electrical stimulation was applied to the centre of the right volar forearm using an epicutaneous pin electrode. This electrode consisted of a circular array of 15 cathodal pins (individual pin diameter: 0.2 mm; length: 1 mm; total array diameter: 10 mm; area: 79 mm^2^), surrounded by a stainless‐steel anode (inner diameter: 20 mm; outer diameter: 40 mm). A constant current stimulator (pulse width: 2 ms; DS7, Digitimer Ltd., Welwyn Garden City, UK) delivered the stimuli. Before electrode placement, participants' forearms were cleaned with isopropyl alcohol. The stimulation site was marked using a circular template matching the electrode's size, with one reference point spaced 1 cm apart along eight radial axes. A corresponding grid was drawn on the left volar forearm to ensure consistency during control assessments. Individual electrical detection thresholds (EDTs) were determined using the method of limits (Gregson 1992). Stimuli were first delivered at an intensity of 0.05 mA, increasing in steps of 0.05 mA until participants reported a distinct sensation. The intensity was then reduced in 0.01 mA increments until the sensation disappeared and subsequently increased again in 0.01 mA increments until the sensation returned. This procedure was repeated three times, and the average of six measurements (three ascending and three descending) was calculated to establish the EDT for each participant. Immediately after calculating participants' EDT, HFS was administered using a pulse generator (D4030; Digitimer Ltd). The protocol consisted of five trains of 100 Hz stimuli, each lasting 1 s, with 10‐s intervals between trains. The stimulation intensity was set at 20 times the individual EDT. Following each train, participants rated their perceived pain intensity in an NRS.

### 2.4 MPS Assessments

Identical MPS assessments were carried out in both forearms at baseline and 30 min post‐HFS using a modified version of the DFNS MPS protocol (Vollert et al. 2016). Three pinprick stimuli with forces of 128, 256 and 512 mN were applied five times each in a pseudo‐randomised sequence within a 1 cm area surrounding the electrode site. To minimise windup effects, the locations of the stimuli were alternated across four quadrants, using the landmarks previously drawn as a guide. After each stimulus, participants rated their perceived pain intensity on an NRS. To control for potential order effects, the sequence in which each forearm was tested (left or right) was counterbalanced across participants but kept consistent within participants.

### 2.5 Procedure

#### 2.5.1 VR Setup

**FIGURE 1:**
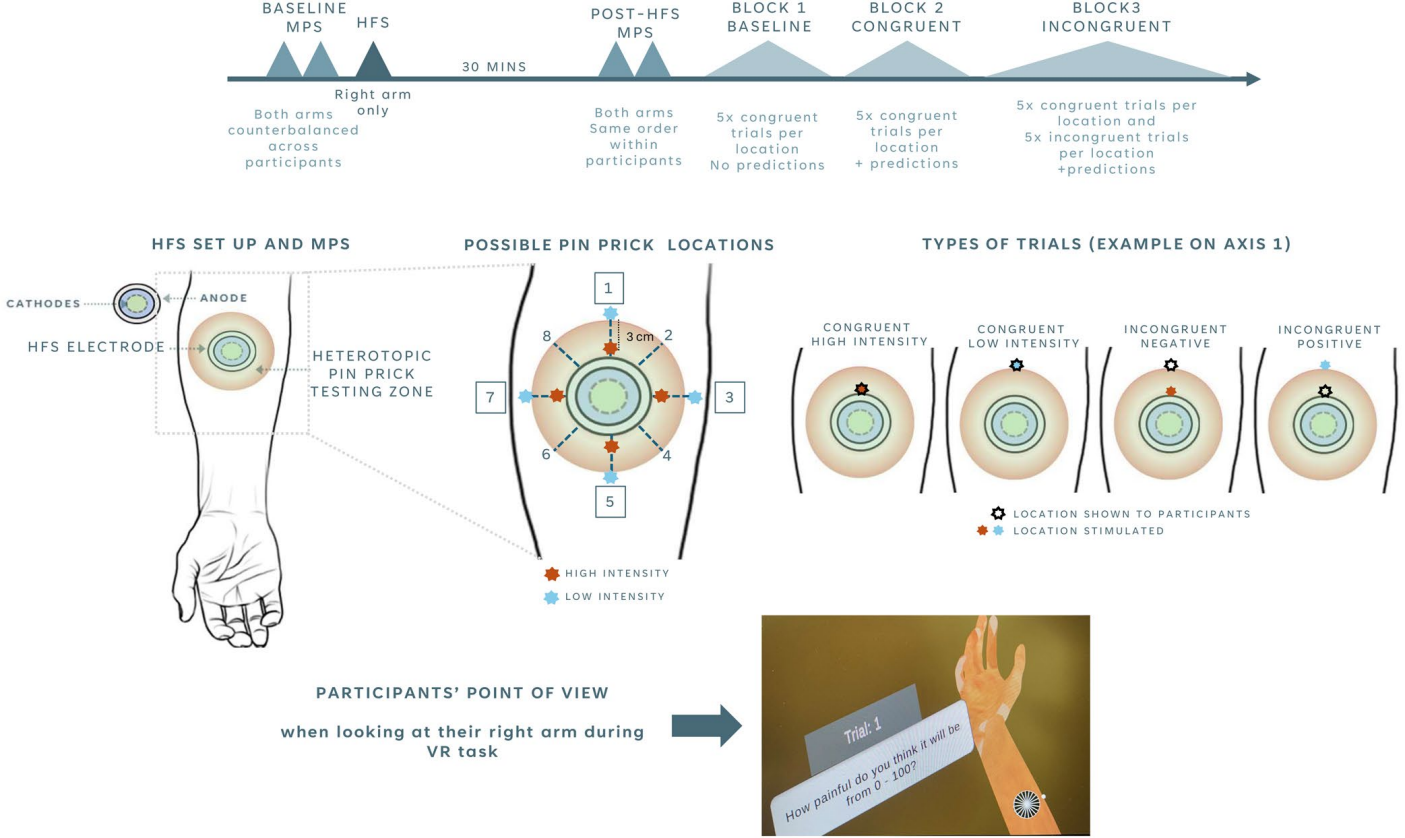
Experimental design (top panel) HFS set up and trial classification (middle panel) and VR set up (bottom panel). All participants attended a single visit and completed an identical protocol. The total duration of the visit was approximately 1.5 h. MPS was defined as the geometric mean of 15 pinprick stimulation (5 × 128 mN, 5 × 256 mN, 5 × 512 mN). Stimuli during the VR task were delivered with a 512 mN pinprick. ‘High‐intensity’ stimuli were delivered 1 cm away from the edge of the electrode placement area, in order to ensure no overlap with the somatotopic area. HFS = high‐frequency stimulation, MPS = mechanical pain sensitivity. HFS set up figure adapted from Medina and Hughes (2024).

Following 30 min post‐HFS MPS assessments, participants were introduced to the VR environment. Seated comfortably with their right arm positioned on a pillow placed on a table in front of them, participants wore an HTC VIVE 2 PRO headset, equipped with an Ultraleap hand‐tracking camera to display a real‐time virtual representation of their arm. The virtual arm included a circle on the location consistent with the HFS electrode placement. Participants were asked to direct their gaze towards their right arm and move their fingers to ensure the virtual representation of their arm responded accurately to their real movements and induced a sense of embodiment of the virtual arm. A total of eight locations were selected for stimulation during the VR task, including four locations on the heterotopic area proximally to the electrode across axes 1, 3, 5 and 7 (from the eight axes originally drawn for MPS assessment) and four distal locations (3 cm away from proximal locations) on the same axes (Figure 1). This placement was informed by our prior mapping work showing that secondary hyperalgesia consistently peaks within 1–2 cm of the heterotopic area surrounding the electrode and diminishes markedly by 4 cm (Medina and Hughes 2024), making the 3 cm distance a reasonable cut‐off to define high‐ and low‐sensitisation sites. All stimuli were delivered with a 512 mN pinprick, which represents a deviation from the original protocol; while our original protocol included 128 mN intensity stimuli, preliminary pilot testing indicated that participants did not report sufficient levels of pain for meaningful data analysis. Consequently, we opted to use the 512 mN pinprick stimulus, which elicited more robust pain ratings. The experimental task included three blocks of trials in total:

*Block 1 (baseline)*: in each trial, a white dot appeared on a location of the virtual arm consistent with the location of the forthcoming stimulus (Figure 1). Approximately 2 s later, Experimenter 1 poked the congruent landmark location on the participant's real right arm. Immediately after, Experimenter 2 displayed a question saying, ‘How painful was the stimulus from 0 to 100?’ next to participants right arm in the virtual environment. Participants answered the question verbally with an NRS score and Experimenter 2 triggered the next trial, where a different dot would appear on the virtual arm. There was a total of 40 consecutive trials (5 trials per stimulus location), presented in randomised order (same order across participants).

*Block 2 (congruent trials)*: trials were similar to those in Block 1, however, an additional prompt was displayed next to participants' right arms in the virtual environment. This prompt read, ‘How painful do you think it will be from 0 to 100?’ appeared immediately after the display of the forthcoming stimulus landmark. Participants provided their predicted pain intensity on an NRS. Approximately 2 s later, Experimenter 2 poked the congruent landmark location on the participant's real right arm. The remainder of the trial sequence followed the same procedure as in Block 1. A total of 40 consecutive trials (5 trials per stimulus location) were presented, maintaining the same order as in Block 1.

*Block 3 (congruent + incongruent trials)*: here, the trial sequence was identical to the one in Block 2; however, the landmark shown on the virtual arm was congruent with the location poked in 50% of the trials. These were referred to as ‘congruent trials’ The remaining trials were referred to as ‘incongruent trials’ Incongruency could be presented in two ways: (i) ‘incongruent negative’ (also referred to as ‘− High’), where participants were shown a low‐intensity landmark but were poked in a high‐intensity landmark within the same axis, resulting in an outcome expected to be more negative than predicted; (ii) ‘incongruent positive’ (also referred to as ‘+ Low’) where participants were shown a high‐intensity landmark but were poked in a low‐intensity landmark within the same axis, resulting in an outcome expected to be more positive than predicted. A total of 80 trials were presented in randomised order (kept consistent across participants), including 40 congruent trials and 40 incongruent trials (20 incongruent negative and 20 incongruent positive). In order to avoid additional sensitisation from temporal summation of pain, no more than two consecutive trials were performed in the same location, with an inter stimulus interval of approximately 5 s.

Participants were instructed to maintain their gaze towards their right arm for the whole duration of the block. Participants were blind to both the force and location of each mechanical stimulus during the PE task, as stimulation was applied while they wore the VR headset and no information about stimulus parameters or experimental conditions was provided. Between blocks, participants were given the option to take short breaks to minimise discomfort from prolonged use of the VR headset. Experimenters 1 and 2 maintained the same roles throughout the study for experimental consistency.

### 2.6 Data Analysis

Analyses were performed using MS Excel and custom‐made Matlab scripts. All statistical tests were considered significant with *α* = 0.05 (Table 1).

**TABLE 1:** Summary of research questions and statistical comparisons.

Analysis purpose	Comparisons	Side	Methods
1. Confirm secondary hyperalgesia	Changes in MPS from baseline	Test and Control arms separately	Paired *t*‐test (MPSbaseline vs. MPSpost‐HFS)
		Test and Control arms separately	One‐sample *t*‐test for Standard MPS changes (MPSpost_HFS − MPSbaseline)/(SDgroup_MPSbaseline)
		Test and Control arms separately	Percentage of change from MPSbaseline to MPSpost‐HFS
		Direct test vs. control arm comparison	Paired *t*‐test for Standard MPS change across arms
2. Confirm stimuli are perceived as more painful within the secondary hyperalgesic area	Changes in MPS from baseline	Test arm only	Averaged across axes: Paired *t*‐test (high‐ vs. low‐intensity trials) within Block 1, Block 2, Block 3 congruent and Block 3 incongruent trials Within each axis: Paired *t*‐test (high‐ vs. low‐intensity trials) within Block 1, Block 2, Block 3 congruent and Block 3 incongruent trials
3. Measure prediction accuracy within each level across conditions	Difference of PEs from 0	Test arm only	One‐sample *t*‐test within each level: Block 2, high‐intensity trials; Block 2, low‐intensity trials; Block 3, congruent high‐intensity trials; Block 3, congruent low‐intensity trials; Block 3, incongruent, high‐intensity trials; Block 3, incongruent, low‐intensity trials
4. Examine whether PEs differ across conditions	Direct comparison of PEs across conditions	Test arm only	Two‐way repeated‐measures ANOVA with condition (Block 2, Block 3 congruent, Block 3 incongruent) and stimulus intensity (high, low) as a within‐subject factors.
5. Examine relation between expectations and pain modulation	Pain score form congruent vs. incongruent trials	Test arm only	Separately for high‐ and low‐intensity trials: Paired *t*‐test between pain scores in Block 2 and pain scores from equivalent incongruent trials in Block 3
	Relationship between extent of pain change and extent of PEs	Test arm only	Separately for high‐ and low‐ intensity trials: Pearson's correlation between PEs and delta pain scores = Block 2 trials—equivalent Block 3 incongruent trials
6. Exploratory analysis: learning effects examination	PEs from first half vs. second half of trials	Test arm only	Block 3 only: Paired *t*‐test (average PEs from first vs. second half of trials) separately for congruent, incongruent negative and incongruent positive trials Block 3 only: Linear regression for average PEs across participants, separately for congruent
	Tendency of PEs to 0	Test arm only	Incongruent negative and incongruent positive trials (i.e., PE as dependent variable and trial number as independent variable)

Abbreviations: HFS = high‐frequency stimulation, MPS = mechanical pain sensitivity, defined as the geometric mean across all raw NRS scores, PE = prediction error, defined as predicted minus perceived NRS score for each trial in Blocks 2 and 3.

#### 2.6.1 HFS Effects on MPS Measures (Both Arms)

MPS indices were defined as the geometric mean of the raw 15 pain ratings of the pinprick stimuli (128, 256 and 512 mN, ×5 times each). Due to one zero rating from a single participant in the control arm, a constant of 0.01 was added to all raw values prior to further calculations, as geometric means are undefined for zero values. MPS changes following HFS were examined via three different strategies; first, raw MPS measures were compared within each arm via paired‐samples *t*‐tests. Secondly, we calculated standard MPS changes from baseline as follows: (MPSpost_HFS − MPSbaseline)/(SDgroup_MPSbaseline) (Medina and Hughes 2024). Thirdly, we computed the percentage of change from baseline MPS to post‐HFS assessment. One‐sample *t*‐tests were performed for standard MPS change and percentage of change scores within each arm to assess significant group changes from 0. Finally, we directly compared standard MPS changes from baseline across arms via a paired‐samples *t*‐test.

#### 2.6.2 Differences in Perceived Pain Between High‐ and Low‐Intensity Stimuli (Test Arm Only)

In order to examine whether stimuli proximal to the HFS electrode (from now on referred to as ‘high‐intensity’ stimuli) are perceived as more painful than stimuli located distally (from now on referred to as ‘low‐intensity’ stimuli), average NRS scores on perceived pain following stimuli across all 4 axes were averaged separately for high‐ and low‐intensity stimuli and compared via a paired‐samples *t*‐test independently for Block 1, Block 2, Block 3 congruent trials; and Block 3 incongruent trials. Identical analyses were performed within each separate axis.

#### 2.6.3 Prediction Accuracy (Test Arm Only)

Prediction errors (PEs) were defined as delta scores between NRS predictions and the following perceived pain NRS scores (PE = NRS_perceived_ − NRS_predicted_) for each trial across three conditions (Block 2, Block 3 congruent, Block 3 incongruent) separately for high‐ and low‐intensity trials, resulting in six levels:

- *Block 2, high‐intensity trials*
- *Block 2, low‐intensity trials*
- *Block 3, congruent, high‐intensity trials*
- *Block 3, congruent, low‐intensity trials*
- *Block 3, incongruent, high‐intensity trials*
- *Block 3, incongruent, low‐intensity trials*

Resulting positive PEs indicated that participants perceived more pain than predicted and vice versa. One‐sample *t*‐tests were performed across PEs within high‐ and low‐intensity stimuli in each group. PEs significantly different from zero denoted low prediction accuracy, while PEs close to zero indicated high prediction accuracy. PE differences across groups were assessed via a two‐way repeated measures analysis of variance (ANOVA) with condition (Block 2, Block 3 congruent, Block 3 incongruent) and stimulus intensity (high, low) as within‐subject factors.

#### 2.6.4 Relationship Between Predicted and Perceived Pain (Test Arm Only)

To test whether congruency of visual cues impacted pain perception, we compared perceived pain NRS scores between Block 2 and incongruent trials in Block 3 (separately for high‐ and low‐intensity trials) by means of a paired‐samples *t*‐test. We chose this comparison because Block 2 contained only congruent trials and therefore served as a ‘true baseline’ whereas congruent and incongruent trials in Block 3 were interleaved. We anticipated that the presence of incongruent trials in Block 3 could influence participants' expectations or perceptual strategies across the block, potentially confounding congruent trial responses. This approach allowed us to isolate the specific impact of introducing incongruency. In order to explore how the extent of these pain changes related to prediction accuracy, Pearson's correlations between PE measures and delta scores calculated from NRS from congruent trials in Block 2 minus incongruent trials in Block 3 were computed, separately for high‐ and low‐intensity stimuli.

#### 2.6.5 Exploratory Analysis: Examination of Learning Effects (Test Arm Only)

Two strategies were adopted to explore whether PEs were updated with time in Block 3; firstly, PEs from the first and second halves of the trials were averaged and compared via a paired samples *t*‐test, separately for congruent, incongruent negative and incongruent positive trials. Secondly, three separate linear regression analyses were performed for averaged PEs across participants in congruent, incongruent negative and incongruent positive trials, with PEs as the dependent variable and trial number as the independent variable.

## 3 Results

### 3.1 Effect of HFS in Mechanical Pain Measures

**FIGURE 2:**
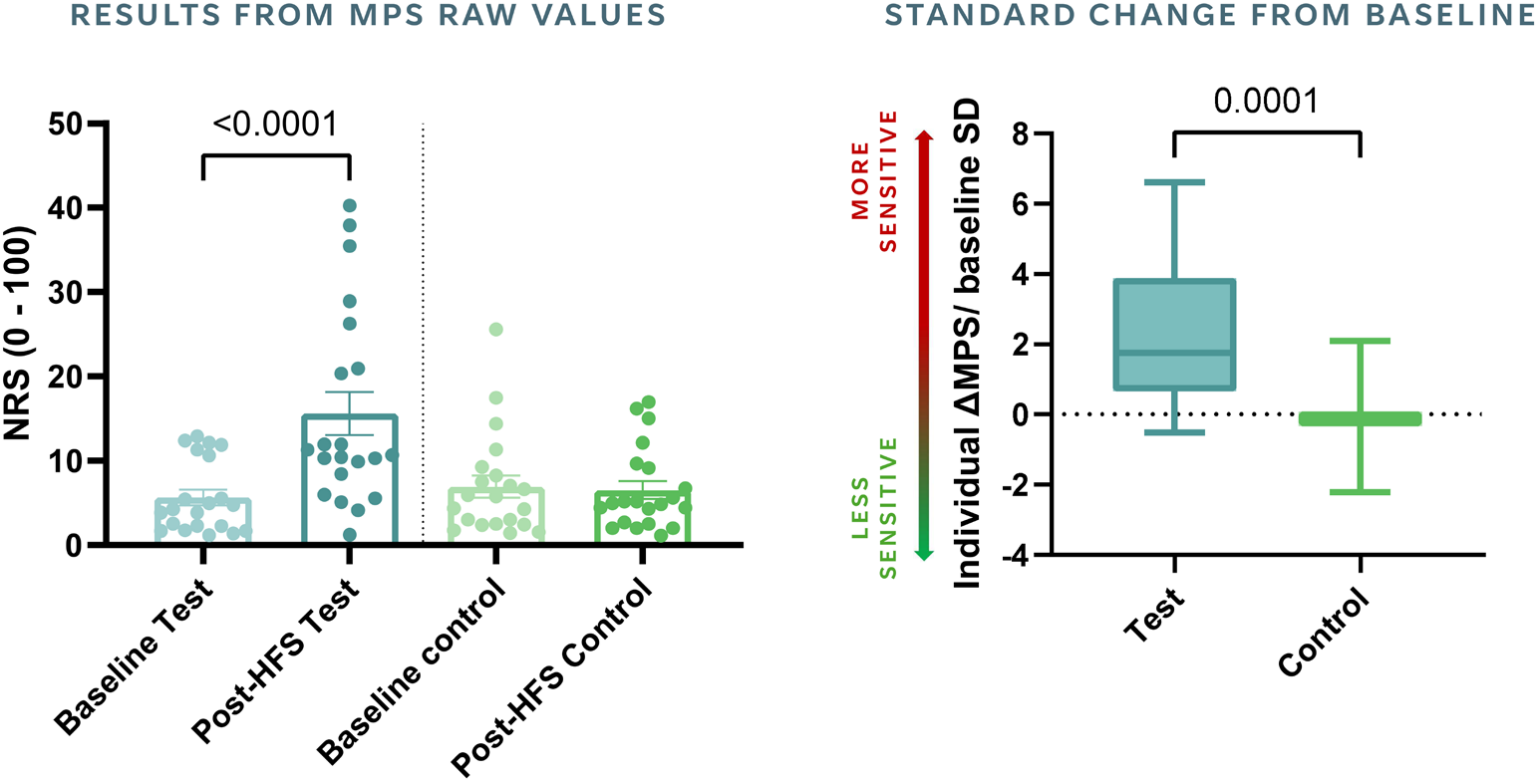
HFS effects on MPS measures. Left panel depicts differences between baseline and post‐HFS assessments in raw MPS values for the test arm (blue) and the control arm (green). Error bars represent the standard error of measurement (SEM). Boxplots (right panel) represent standard changes in MPS measures from baseline for the test arm (blue) and the control arm (green). Standard MPS changes were significantly greater than zero in the test arm only, indicating the presence of secondary hyperalgesia in a constricted area surrounding the HFS site. MPS = mechanical pain sensitivity, NRS = numerical rating scale (ranged from 0 ‘no pain at all’ to 100 ‘worst pain imaginable’).

Paired‐samples *t*‐test indicated that MPS significantly increased post‐HFS within participants in the test arm (mean MPS_baseline_ (SD) = 5.65 (4.26), mean MPS_post‐HFS_ (SD) = 15.59 (11.67), *t*_(19)_ = 5.09 *p* < 0.001). One‐sample *t*‐tests for standard MPS changes from baseline within each arm revealed a significant group increase in MPS measures for the test arm (*t*_(19)_ = 5.09 *p* < 0.001), averaging 2.33 standard deviations (Figure 2), and a significant 240% increase in MPS measures (*t*_(19)_ = 4.45 *p* < 0.001). There were no significant changes across any of the MPS indices for measures from the control arm. Paired‐samples *t*‐test indicated that standard MPS changes from baseline were significantly higher in the test arm than in the control arm (*t*_(19)_ = 4.72 *p* < 0.001).

### 3.2 Differences in Perceived Pain Between High‐ and Low‐Intensity Stimuli

**FIGURE 3:**
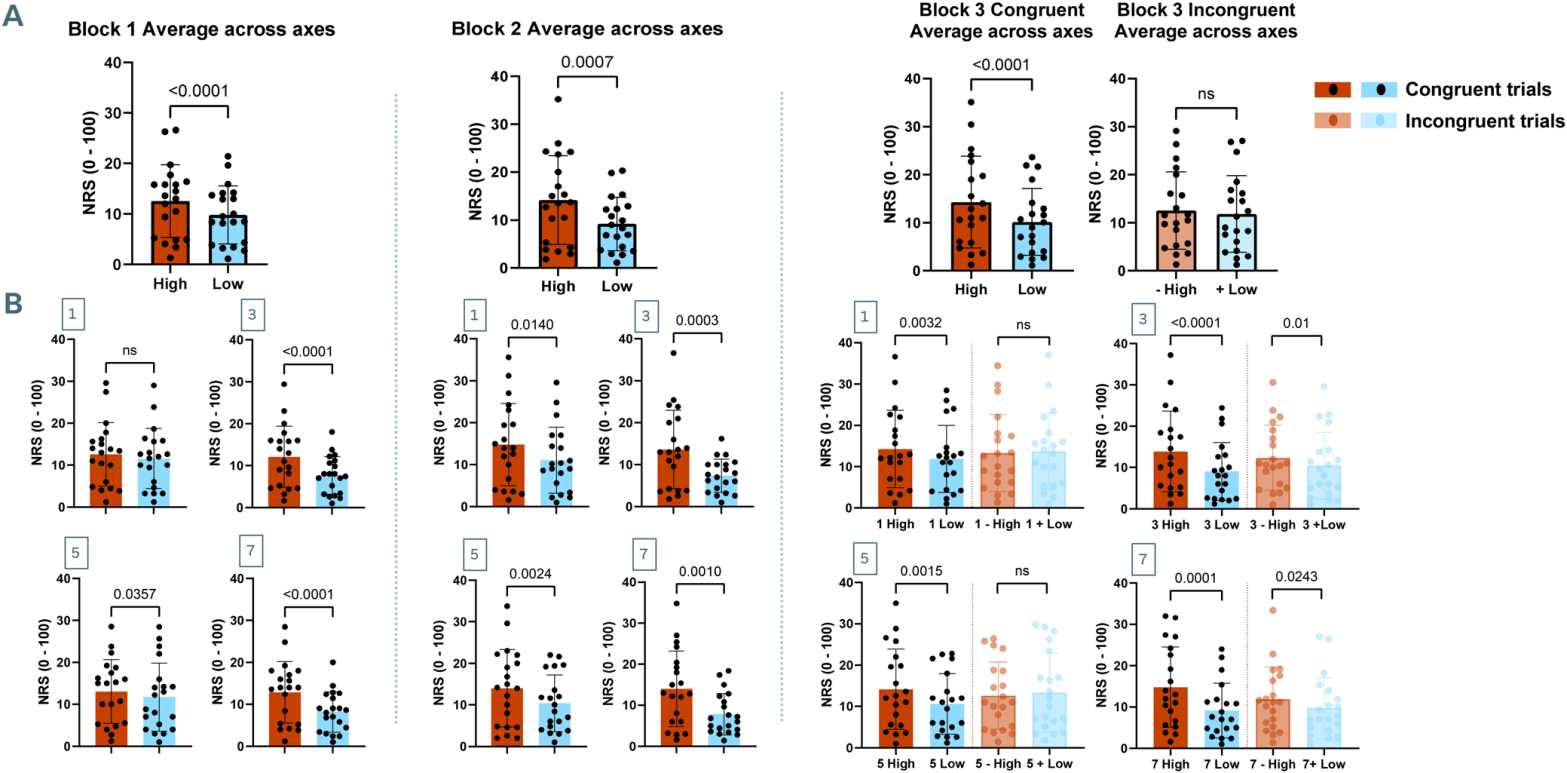
Summary of perceived pain intensity results across blocks for high‐ (red) and low‐ (blue) intensity stimuli. (A) Perceived pain rating results for high‐ and low‐intensity stimuli across all axes. Lighter colour bar plots represent results from high‐ and low‐intensity stimuli preceded by incongruent visual cues. (B) Perceived pain rating results for high‐ and low‐intensity trials within each axis. Paired‐samples *t*‐tests indicated that high‐intensity stimuli were perceived as significantly more painful than low‐intensity trials in most congruent trials, except for axis 1 in Block 1 (second row). There were no significant differences between high‐ and average low‐intensity incongruent trials for average NRS, axis 1 and axis 5. Error bars represent the SEM. NRS = numerical rating scale (from 0 ‘no pain at all’ to 100 ‘worst pain imaginable’).

Paired‐samples *t*‐test revealed that stimuli located proximally to the HFS electrode (high‐intensity stimuli) were perceived on average as significantly more painful than stimuli located distally (low‐intensity stimuli) in Block 1, Block 2 and Block 3 ‘congruent trials’ (*t*_(19)_ = 5.80 *p* < 0.001, *t*_(19)_ = 4.03 *p* < 0.001, *t*_(19)_ = 5.30 *p* < 0.001, respectively). This was also the case when considering stimuli within each individual axis, except for axis 1 during Block 1 (Figure 3). Results for Block 3 ‘incongruent trials’ revealed that on average, high‐ and low‐intensity trials were no longer perceived as significantly different, although significance was still reached when considering axis 3 and axis 7 independently (*t*_(19)_ = 2.86 *p* = 0.01, *t*_(19)_ = 2.44 *p* = 0.02, respectively).

### 3.3 PEs and Congruency

**FIGURE 4:**
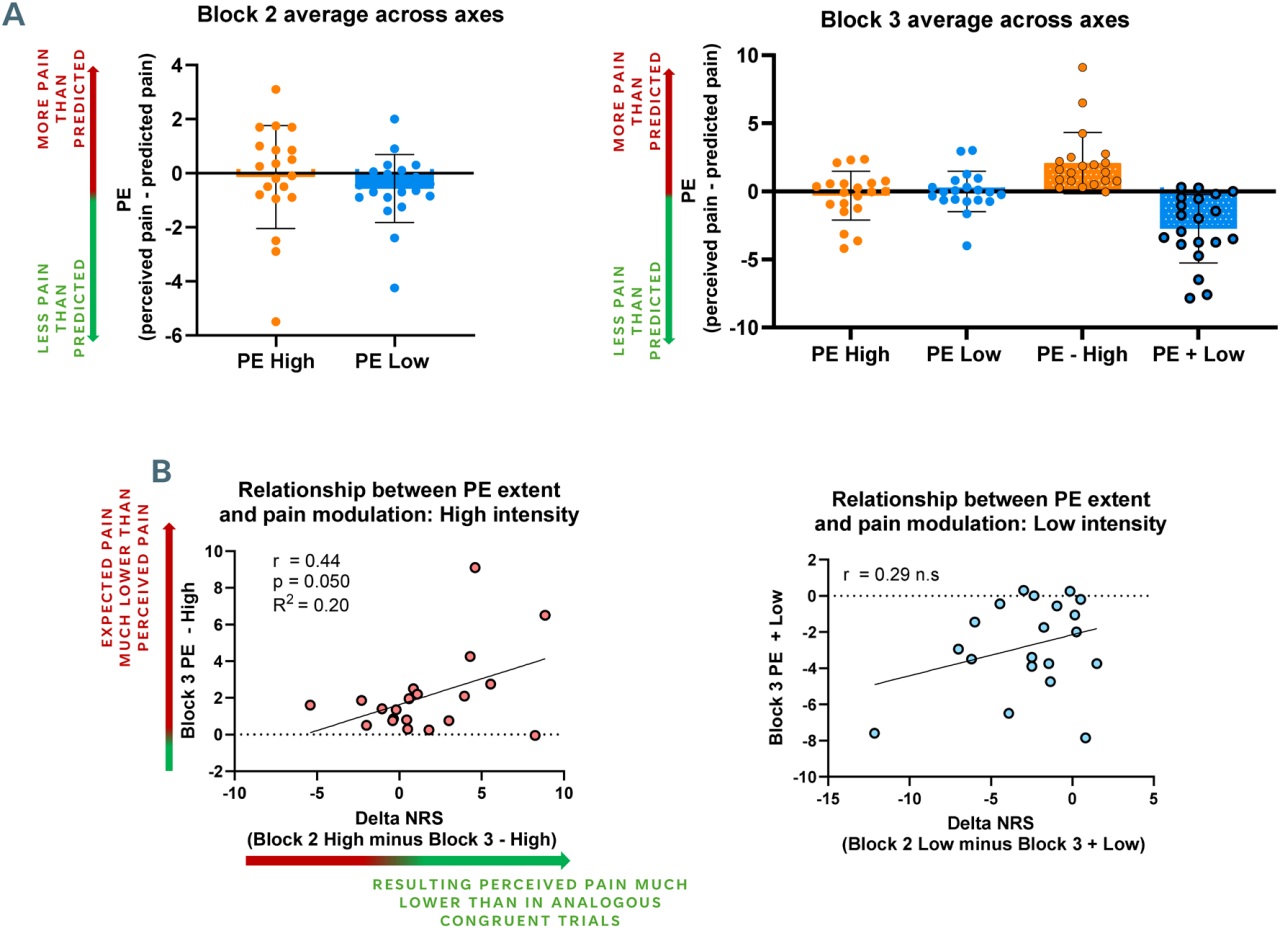
Summary of PE results. (A) Group level results for PEs following high‐ (orange) and low‐ (blue) intensity trials during Block 2 (left panel) and Block 3 (right panel). PEs across all congruent trials were not significantly different from zero, indicating high prediction accuracy. PEs for incongruent negative trials were significantly greater than zero, reflecting significantly greater pain than expected, and PEs for incongruent positive trials were significantly lower than zero, reflecting significantly lower pain than expected. (B) Left panel: Pearson's correlation between delta scores (resulting from subtracting NRS scores from high‐intensity trials in Block 2 minus NRS scores from analogous incongruent trials, *x* axis) and PEs for incongruent negative stimuli (*y* axis). A significant positive correlation was identified. Right panel: Homologous scatter plot for low‐intensity trials. No significant correlation was found. NRS = numerical rating scale, n.s = not significant, PE = prediction error.

One‐sample *t*‐tests for PEs within high‐ and low‐intensity trials for each group (Block 2, Block 3 congruent trials, Block 3 incongruent trials) revealed no significant differences from zero for high‐ and low‐intensity stimuli in Block 2 and Block 3 congruent trials (Figure 4). PEs were significantly greater than zero for incongruent negative trials (− High), indicating that participants perceived on average significantly more pain than predicted (*t*_(19)_ = 4.15 *p* < 0.001). Conversely, PEs were significantly lower than zero for incongruent positive trials (+ Low), reflecting that participants perceived less pain than predicted during these stimuli (*t*_(19)_ = 4.86 *p* < 0.001). To further test the effect of congruency, we conducted additional post hoc paired‐samples *t*‐tests comparing incongruent and congruent trials within each intensity condition. These revealed significantly greater PEs for incongruent compared to congruent trials for both high‐ and low‐intensity stimuli (all *p* < 0.01).

One‐way ANOVA revealed a main effect of stimulus intensity (*F*_(2,38)_ = 19.02 *p* < 0.001, partial *η* ^2^ = 0.48) with PEs for high‐intensity trials being significantly greater than low‐intensity trials across conditions (mean difference = 1.64 *p* < 0.001) and a significant interaction effect (*F*_(2,38)_ = 17.96 *p* < 0.001, partial *η* ^2^ = 0.48), driven by significant PE differences between high‐ and low‐intensity trials within the Block 3 incongruent block but not in the others (Figure 4).

### 3.4 PEs and Pain Modulation

We carried out a paired‐samples *t*‐test to examine whether stimulus congruency influenced perceived pain intensity. There was a significant difference between congruent trials in Block 2 and analogous incongruent trials in Block 3. Concretely, low‐intensity stimuli in Block 2 (i.e., when a congruent visual cue was provided) were perceived as less painful than in Block 3 incongruent trials (i.e., when an incongruent, high‐intensity visual cue was provided), *t*_(19)_ = 3.56 *p* = 0.002. We found no significant difference for the perception of high‐intensity stimuli in Block 2 and in Block 3 incongruent trials. Pearson's correlations revealed that this difference in perceived pain across blocks correlated positively with PEs for incongruent trials of the same stimulus intensity for high‐intensity trials (Figure 4B). This is, participants who predicted much lower pain than perceived in incongruent negative trials still reported lower pain than in analogous congruent trials and vice versa. We found no significant correlation for low‐intensity stimuli.

### 3.5 Learning Effects

**FIGURE 5:**
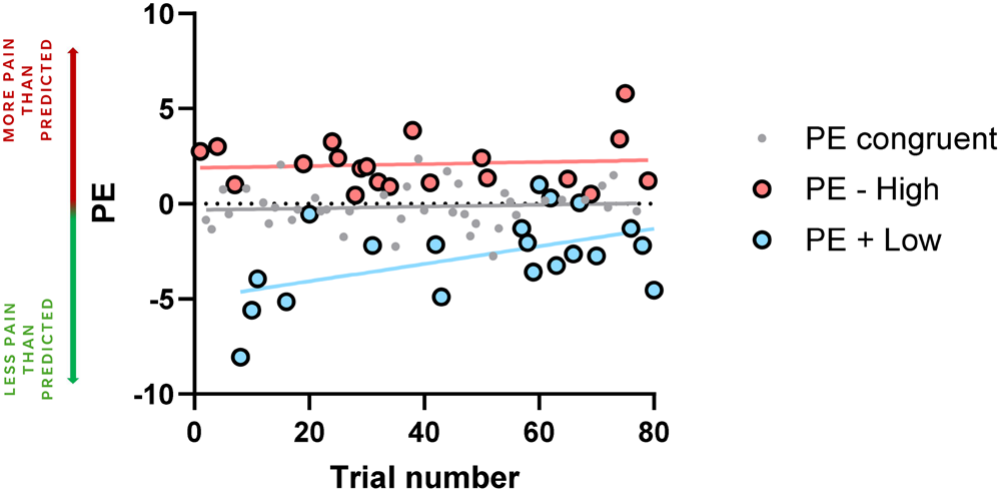
Exploratory linear regression analyses on potential learning effects on PEs in Block 3. PEs significantly tended to zero as a function of trial number in the case of incongruent positive trials (+ Low, blue‐coloured dots), but not in the case of incongruent negative (− High, salmon‐coloured dots) and congruent trials (grey coloured dots). PE = prediction error.

While our first exploratory analysis revealed no significant differences between average PEs from the first and second halves of trials of each group (Block 3 congruent, Block 3 incongruent negative, Block 3 incongruent positive), linear regression analyses indicated that there was a significant tendency to zero (i.e., no error of prediction) for PEs in incongruent low‐intensity trials as a function of trial number (*F*_(1,18)_ = 6.25 *p* = 0.02, *R* ^2^ = 0.26, *Y* = 0.046*X* − 5.003). PEs were not significantly predicted by trial number for congruent or incongruent high‐intensity trials (Figure 5).

## 4 Discussion

This study introduces a novel VR‐based approach to examine the relationship between PEs and mechanical secondary hyperalgesia within the predictive coding framework of pain in healthy individuals. All preregistered hypotheses were confirmed: HFS successfully induced secondary hyperalgesia, as evidenced by increased MPS in the heterotopic area from HFS stimulation; a priori defined high‐ and low‐intensity stimulation sites were correctly perceived as more and less painful, respectively; and PEs were effectively generated by manipulating participants' pain expectations, impacting subsequent pain perception. Crucially, exploratory analyses revealed adaptive updating of PEs (tending towards zero) for low‐intensity stimuli (i.e., stimuli on non‐sensitised areas). In contrast, high‐intensity stimuli (i.e., stimuli on sensitised areas) showed persistent PEs, which may reflect disrupted updating of predictions, a pattern that has been proposed as a feature of maladaptive coding in chronic pain (Castejón et al. 2024; Hechler et al. 2016; Hoskin and Talmi 2023; Song et al. 2021; Traxler et al. 2022). This underscores the potential of this model to assess pharmacological, cognitive and psychological interventions in predictive coding under controlled sensitised states.

HFS successfully elicited secondary mechanical hyperalgesia, consistent with previous findings employing the same adaptation of the DFNS MPS protocol (Medina and Hughes 2024) and similar mechanical assessments (Fawsitt‐Jones et al. 2024; van den Broeke et al. 2011; Vo and Drummond 2013, 2016). This phenomenon has been described in the literature as a manifestation of long‐term potentiation of synapses between primary afferent C‐fibres and second level afferent neurons in the superficial dorsal horn of the spinal cord (Klein et al. 2004; Magerl et al. 2018; Meijs et al. 2025; Ruscheweyh et al. 2011; van den Broeke and Mouraux 2014), and it is therefore considered a robust model of CS (Leone et al. 2021; Torta et al. 2023; van Amerongen et al. 2016; Van den Broeke et al. 2016; Vollert et al. 2018). Since CS is thought to be an important neurophysiological mechanism contributing to the establishment and maintenance of chronic pain, especially in the case of inflammatory and neuropathic pain (Fornasari 2012; Harte et al. 2018; Volcheck et al. 2023; Woolf 2011), the HFS model has been used to assess the influence of top‐down cognitive components of pain perception during experimentally controlled sensitised states, such as expectations (Jaltare et al. 2024; van den Broeke et al. 2014), fear and empathy (Torta et al. 2023), or cognitive demand (Torta et al. 2020) as well as to provide therapeutic biomarkers (Medina and Hughes 2024; Mehesz et al. 2021; Ruscheweyh et al. 2011). However, to the best of our knowledge, this is the first attempt to characterise patterns of PE coding and pain inference during CS in healthy individuals using this model. Participants perceived the so‐called ‘high‐intensity’ stimuli as significantly more painful than ‘low‐intensity’ stimuli (Medina and Hughes 2024). Since the volar forearm has a relatively uniform sensory innervation through median and ulnar nerves (Borges and Souza 2021), these results were likely to arise due to differences in secondary hyperalgesia intensity and not sensitivity variations. Moreover, 11 out of 12 within‐axis comparisons for congruent trials were significant, suggesting that despite some individual variability, there was limited heterogeneity across axes, justifying the use of averaged responses for clarity and interpretability.

Our protocol successfully induced prediction PEs by manipulating the congruency between the shown stimulus location and the actual stimulus location. These PEs occurred in the hypothesised direction: participants perceived greater pain when stimulated at a high‐intensity landmark while shown a low‐intensity landmark, and less pain when the reverse was true. Importantly, expectations influenced subsequent pain perception; low‐intensity stimuli were rated as more painful when participants anticipated high‐intensity pain, consistent with the principles of nocebo hyperalgesia (Bräscher and Witthöft 2019; Camerone et al. 2022; Hechler et al. 2016; Tracey 2010). This occurred despite participants making highly accurate predictions (as shown in congruent trials), indicating that incongruent trials involved strong and specific expectations; this is conceptually distinct from paradigms using ambiguous cues (Pavy et al. 2024), where unpredictability itself modulates pain via imprecise expectations.

In contrast, high‐intensity stimuli were not rated as significantly less painful when participants anticipated low‐intensity pain compared to congruent expectations. This finding diverges from the principles of placebo analgesia (Büchel et al. 2014; Camerone et al. 2022; Jaltare et al. 2024; Tracey 2010), where positive expectations are thought to engage endogenous opioid pathways that dampen nociceptive input (Benedetti and Amanzio 1997; Colloca et al. 2013; Petrovic et al. 2002; Wager et al. 2007). It is reasonable to argue that this discrepancy could be attributed to the influence of CS within the peak hyperalgesic area, where amplified nociceptive drive overrides top‐down modulatory mechanisms of placebo analgesia (Lim et al. 2020). Further, previous evidence suggests that the direction of the mismatch is encoded by differential brain mechanisms (Ploghaus et al. 2000), which may be differently affected by CS.

There was nevertheless a significant correlation between PEs and the extent of placebo analgesia‐like effects during high‐intensity trials, underscoring the critical role of expectations in pain modulation, even in the presence of CS. Conversely, during low‐intensity stimulation, PEs appeared to play a diminished role in pain modulation, consistent with previous findings in thermal pain (Strube et al. 2021). This is also reflected in our data by a lack of significant correlation between PEs and pain modulation during low‐intensity trials. These findings demonstrate how CS may alter the interplay between predictive coding and nociceptive processing, providing a mechanistic explanation for the persistence of maladaptive pain predictions in chronic pain (Castejón et al. 2024; Eckert et al. 2022). Follow‐up studies may explore how trait‐like bias, such as pessimistic tendencies during pain prediction (Hoskin et al. 2019) or general fear of pain (Aslaksen and Lyby 2015; Feldhaus et al. 2021; Thomaidou et al. 2021), interact with the effects observed in the present study. Such insights have profound implications for understanding pain perception and developing strategies to recalibrate maladaptive predictive coding in sensitised states.

The contrasting patterns of PE adaptation over time observed offer insights into how central CS disrupts predictive coding of pain. When participants were shown a low‐intensity landmark while poked in a highly sensitised area, PEs remained constant across trials, suggesting that the heightened nociceptive input from sensitised regions may impair the brain's ability to update its predictions. This rigidity suggests that in CS, the brain prioritises the amplified nociceptive signals over sensory feedback, preventing error correction (Büchel et al. 2014; Mulders et al. 2023; Roy et al. 2014; Seymour and Mancini 2020). Conversely, when participants were shown a landmark in a highly sensitised area while poked in a less sensitised area, PEs diminished across trials, reflecting normal adaptive learning to maximise precision, according to the principles of predictive processing (Geuter et al. 2017; Hoskin et al. 2019; Tabor and Burr 2019) and reinforcement learning (Seymour 2019). These findings imply that the presence of CS may alter the balance between prior expectations and sensory updates, leading to a maladaptive persistence of predictions in sensitised regions (Vlaeyen 2015). This phenomenon may mirror mechanisms underlying chronic pain, where excessive nociceptive signals perpetuate inaccurate predictions and hinder the resolution of pain, contributing to its persistence (Nees and Becker 2018; Ramne and Sensinger 2024).

This model provides a controlled framework to investigate how CS affects predictive coding and pain perception, with potential applications in both research and clinical settings. Moving forward, it could be used to assess how different interventions (pharmacological, psychological, or cognitive) impact predictive coding in the presence of CS (Bishop et al. 2018; Jepma et al. 2022). For example, treatments aimed at reducing hyperalgesia or modulating expectations (e.g., cognitive‐behavioural therapy, mindfulness‐based stress reduction) (Karoly 2021; Tsai et al. 2024) or promoting placebo analgesia (e.g., hypnosis, placebo interventions) (Schenk et al. 2017) could be evaluated by examining changes in PEs and pain modulation patterns. In clinical contexts, this VR model could help identify individuals with maladaptive predictive coding, offering a tailored approach to treatment (Strigo et al. 2024; Wager et al. 2011). Disentangling the roles of nociceptive input and expectation updating could provide insights into maladaptive mechanisms linked to chronic pain but not fully explained by the presence of CS (Velasco et al. 2024). Additionally, integrating this model into neuroimaging or neurophysiological studies could reveal the neural circuits disrupted by CS and how they respond to therapeutic interventions (Chen 2023; Mulders et al. 2023; Nickel et al. 2022; Roy et al. 2014). Finally, the model could inform the design of VR‐based rehabilitation tools (Matthie et al. 2022; Medina et al. 2024), using controlled manipulations of visual feedback to retrain the brain's predictive coding processes and reduce maladaptive pain perceptions.

This study also presents limitations that should be acknowledged. Firstly, the unbalanced female‐to‐male ratio could have introduced potential gender‐related bias regarding pain estimation (Zhang et al. 2021) and pain perception mechanisms during secondary hyperalgesia (Asghar et al. 2015). Secondly, the absence of a baseline VR task assessment prior to HFS, or of similar assessments in the control arm following HFS, limits our ability to disentangle the specific effects of HFS‐induced central sensitisation on predictive coding from participants' baseline predictive processes. While the focus of this study was to assess the viability of the VR task during HFS, control assessments for the VR task may be implemented in future iterations of this model. Finally, it is worth considering that while participants might have consciously realised the mismatch between the poked location and the visual cue in incongruent trials, it may not necessarily be a critical limitation for studying PEs; Predictive Coding Theory posits that PEs arise automatically when sensory inputs deviate from predictions, regardless of conscious awareness (Rowe et al. 2020). Therefore, even if participants consciously recognise the mismatch, the underlying neural mechanisms driving PEs are likely still engaged. Nonetheless, these considerations highlight the need for further studies to validate and extend our findings.

In summary, this study presents a novel VR‐based approach to explore the interplay between PEs and secondary mechanical hyperalgesia in healthy individuals. We successfully demonstrated that expectations influence pain perception in sensitised and non‐sensitised areas differently. Importantly, the findings suggest that in sensitised states, PEs persist, potentially reflecting maladaptive predictive coding mechanisms similar to those implicated in chronic pain. Therefore, we propose this model as a potentially viable candidate to provide a controlled framework to study interventions targeting maladaptive PE mechanisms. By enabling precise manipulation of expectations and nociceptive input, this new experimental approach can help to inform the development of tailored VR‐based rehabilitation strategies targeting PE mechanisms during chronic pain. These findings pave the way for future research to refine the model, validate its clinical relevance and explore its applications in pain management and rehabilitation.

## Conflicts of Interest

The authors declare no conflicts of interest.

## Data Availability

Data are available upon request.

## References

[ejp70113-bib-0001] Asghar, M. S. , M. P. Pereira , M. U. Werner , J. Mårtensson , H. B. Larsson , and J. B. Dahl . 2015. “Secondary Hyperalgesia Phenotypes Exhibit Differences in Brain Activation During Noxious Stimulation.” PLoS One 10, no. 1: e0114840. 25615578 10.1371/journal.pone.0114840 PMC4304709

[ejp70113-bib-0002] Aslaksen, P. M. , and P. S. Lyby . 2015. “Fear of Pain Potentiates Nocebo Hyperalgesia.” Journal of Pain Research 8: 703–710. 10.2147/JPR.S91923. 26491370 PMC4608595

[ejp70113-bib-0003] Bannister, K. , and S. Hughes . 2023. “One Size Does Not Fit All: Towards Optimising the Therapeutic Potential of Endogenous Pain Modulatory Systems.” Pain 164, no. 1: e5–e9. 10.1097/j.pain.0000000000002697. 35594517 PMC9756434

[ejp70113-bib-0004] Baron, R. , G. Hans , and A. H. Dickenson . 2013. “Peripheral Input and Its Importance for Central Sensitization.” Annals of Neurology 74, no. 5: 630–636. 10.1002/ana.24017. 24018757

[ejp70113-bib-0005] Benedetti, F. , and M. Amanzio . 1997. “The Neurobiology of Placebo Analgesia: From Endogenous Opioids to Cholecystokinin.” Progress in Neurobiology 52, no. 2: 109–125. 10.1016/S0301-0082(97)00006-3. 9185235

[ejp70113-bib-0006] Bishop, M. D. , J. M. Beneciuk , M. Alappattu , and J. E. Bialosky . 2018. “Experience Matters: Post‐Intervention Expectations Predict Post‐Intervention Hypoalgesia.” Physical Medicine and Rehabilitation Research 3, no. 4: 1–6.31172033

[ejp70113-bib-0007] Borges, Á. V. R. M. , and S. A. L. Souza . 2021. “Anatomy of the Nerves, Vessels, and Muscular Compartments of the Forearm, as Revealed by High‐Resolution Ultrasound. Part 1: Overall Structure and Forearm Compartments.” Radiologia Brasileira 54, no. 6: 388–397. 10.1590/0100-3984.2021.0030. 34866699 PMC8630951

[ejp70113-bib-0008] Bräscher, A.‐K. , and M. Witthöft . 2019. “Nocebo Hyperalgesia Induced by Implicit Conditioning.” Journal of Behavior Therapy and Experimental Psychiatry 64: 106–112. 10.1016/j.jbtep.2019.03.006. 30952053

[ejp70113-bib-0009] Büchel, C. , S. Geuter , C. Sprenger , and F. Eippert . 2014. “Placebo Analgesia: A Predictive Coding Perspective.” Neuron 81, no. 6: 1223–1239. 10.1016/j.neuron.2014.02.042. 24656247

[ejp70113-bib-0010] Camerone, E. M. , G. Tosi , and D. Romano . 2022. “The Role of Pain Expectancy and Its Confidence in Placebo Hypoalgesia and Nocebo Hyperalgesia.” Pain. https://journals.lww.com/pain/fulltext/9900/the_role_of_pain_expectancy_and_its_confidence_in.785.aspx. 10.1097/j.pain.0000000000003495 PMC12168809 39679646

[ejp70113-bib-0011] Castejón, J. , F. Chen , A. Yasoda‐Mohan , C. Ó Sé , and S. Vanneste . 2024. “Chronic Pain—A Maladaptive Compensation to Unbalanced Hierarchical Predictive Processing.” NeuroImage 297: 120711. 10.1016/j.neuroimage.2024.120711. 38942099

[ejp70113-bib-0012] Chen, Z. S. 2023. “Hierarchical Predictive Coding in Distributed Pain Circuits.” Frontiers in Neural Circuits 17: 1073537. 36937818 10.3389/fncir.2023.1073537 PMC10020379

[ejp70113-bib-0013] Clark, R. 2022. Understanding Hand Interactions and Mid‐Air Haptic Responses Within Virtual Reality and Beyond. Bournemouth University.

[ejp70113-bib-0014] Colloca, L. , R. Klinger , H. Flor , and U. Bingel . 2013. “Placebo Analgesia: Psychological and Neurobiological Mechanisms.” Pain 154, no. 4: 511–514. https://journals.lww.com/pain/fulltext/2013/04000/placebo_analgesia__psychological_and.3.aspx. 23473783 10.1016/j.pain.2013.02.002 PMC3626115

[ejp70113-bib-0015] Eckert, A.‐L. , K. Pabst , and D. M. Endres . 2022. “A Bayesian Model for Chronic Pain.” Frontiers in Pain Research 3: 966034. 10.3389/fpain.2022.966034. 36303889 PMC9595216

[ejp70113-bib-0016] Fawsitt‐Jones, H. , J. Vollert , O. O'Daly , et al. 2024. “Reliability of Quantitative Sensory Testing in the Assessment of Somatosensory Function After High‐Frequency Stimulation–Induced Sensitisation of Central Nociceptive Pathways.” Pain 165, no. 4: 941–950. https://journals.lww.com/pain/fulltext/2024/04000/reliability_of_quantitative_sensory_testing_in_the.19.aspx. 37878469 10.1097/j.pain.0000000000003093

[ejp70113-bib-0017] Feldhaus, M. H. , B. Horing , C. Sprenger , and C. Büchel . 2021. “Association of Nocebo Hyperalgesia and Basic Somatosensory Characteristics in a Large Cohort.” Scientific Reports 11, no. 1: 762. 10.1038/s41598-020-80386-y. 33436821 PMC7804006

[ejp70113-bib-0018] Fornasari, D. 2012. “Pain Mechanisms in Patients With Chronic Pain.” Clinical Drug Investigation 32, no. 1: 45–52. 10.2165/11630070-000000000-00000. 22356223

[ejp70113-bib-0019] Geuter, S. , S. Boll , F. Eippert , and C. Büchel . 2017. “Functional Dissociation of Stimulus Intensity Encoding and Predictive Coding of Pain in the Insula.” eLife 6: e24770. 10.7554/eLife.24770. 28524817 PMC5470871

[ejp70113-bib-0020] Gregson, R. A. 1992. “The Psychophysical Method of Limits: What Actually Happens in a Nonlinear Context?” British Journal of Mathematical and Statistical Psychology 45, no. 2: 177–196.

[ejp70113-bib-0021] Harte, S. E. , R. E. Harris , and D. J. Clauw . 2018. “The Neurobiology of Central Sensitization.” Journal of Applied Biobehavioral Research 23, no. 2: e12137. 10.1111/jabr.12137. PMC6251410 30479469

[ejp70113-bib-0022] Haythornthwaite, J. A. , C. M. Campbell , and R. R. Edwards . 2024. “When Thinking About Pain Contributes to Suffering: The Example of Pain Catastrophizing.” Pain 165, no. 11S: S68–S75. 10.1097/j.pain.0000000000003372. 39560417 PMC11581624

[ejp70113-bib-0023] Hechler, T. , D. Endres , and A. Thorwart . 2016. “Why Harmless Sensations Might Hurt in Individuals With Chronic Pain: About Heightened Prediction and Perception of Pain in the Mind.” Frontiers in Psychology 7. 10.3389/fpsyg.2016.01638. PMC5078757 27826271

[ejp70113-bib-0024] Hollander, M. d. , J. R. de Jong , S. Volders , M. E. J. B. Goossens , R. J. E. M. Smeets , and J. W. S. Vlaeyen . 2010. “Fear Reduction in Patients With Chronic Pain: A Learning Theory Perspective.” Expert Review of Neurotherapeutics 10, no. 11: 1733–1745. 10.1586/ern.10.115. 20977330

[ejp70113-bib-0025] Hoskin, R. , C. Berzuini , D. Acosta‐Kane , W. El‐Deredy , H. Guo , and D. Talmi . 2019. “Sensitivity to Pain Expectations: A Bayesian Model of Individual Differences.” Cognition 182: 127–139. 10.1016/j.cognition.2018.08.022. 30243037

[ejp70113-bib-0026] Hoskin, R. , and D. Talmi . 2023. “Adaptive Coding of Pain Prediction Error in the Anterior Insula.” European Journal of Pain 27, no. 6: 766–778. 10.1002/ejp.2093. 36799445

[ejp70113-bib-0027] Jaltare, K. P. , E. Meyers , and D. M. Torta . 2024. “The Role of Pain Expectations in the Development of Secondary Pinprick Hypersensitivity: Behavioral‐Neurophysiological Evidence and the Role of Pain‐Related Fear.” Journal of Pain 25, no. 9: 104567. 10.1016/j.jpain.2024.104567. 38750990

[ejp70113-bib-0028] Jepma, M. , M. Roy , K. Ramlakhan , M. van Velzen , and A. Dahan . 2022. “Different Brain Systems Support Learning From Received and Avoided Pain During Human Pain‐Avoidance Learning.” eLife 11: e74149. 10.7554/eLife.74149. 35731646 PMC9217130

[ejp70113-bib-0029] Karoly, P. 2021. “How Pain Shapes Depression and Anxiety: A Hybrid Self‐Regulatory/Predictive Mind Perspective.” Journal of Clinical Psychology in Medical Settings 28, no. 2: 201–211. 10.1007/s10880-019-09693-5. 31897919

[ejp70113-bib-0030] Klein, T. , W. Magerl , H.‐C. Hopf , J. Sandkühler , and R.‐D. Treede . 2004. “Perceptual Correlates of Nociceptive Long‐Term Potentiation and Long‐Term Depression in Humans.” Journal of Neuroscience 24, no. 4: 964–971. 14749441 10.1523/JNEUROSCI.1222-03.2004 PMC6729815

[ejp70113-bib-0031] Knaggs, R. 2016. “Personalised Medicine and Medicines Optimisation.” British Journal of Pain 10, no. 4: 167. 10.1177/2049463716671224. 27867505 PMC5102102

[ejp70113-bib-0032] Latremoliere, A. , and C. J. Woolf . 2009. “Central Sensitization: A Generator of Pain Hypersensitivity by Central Neural Plasticity.” Journal of Pain 10, no. 9: 895–926. 10.1016/j.jpain.2009.06.012. 19712899 PMC2750819

[ejp70113-bib-0033] Leone, C. , A. Di Lionardo , G. Di Pietro , et al. 2021. “How Different Experimental Models of Secondary Hyperalgesia Change the Nociceptive Flexion Reflex.” Clinical Neurophysiology 132, no. 12: 2989–2995. 10.1016/j.clinph.2021.08.018. 34715423

[ejp70113-bib-0034] Lersch, F. E. , F. C. S. Frickmann , R. D. Urman , et al. 2023. “Analgesia for the Bayesian Brain: How Predictive Coding Offers Insights Into the Subjectivity of Pain.” Current Pain and Headache Reports 27, no. 11: 631–638. 10.1007/s11916-023-01122-5. 37421540 PMC10713672

[ejp70113-bib-0035] Lim, M. , C. O'Grady , D. Cane , et al. 2020. “Threat Prediction From Schemas as a Source of Bias in Pain Perception.” Journal of Neuroscience 40, no. 7: 1538–1548. 10.1523/jneurosci.2104-19.2019. 31896672 PMC7044740

[ejp70113-bib-0036] Magerl, W. , N. Hansen , R.‐D. Treede , and T. Klein . 2018. “The Human Pain System Exhibits Higher‐Order Plasticity (Metaplasticity).” Neurobiology of Learning and Memory 154: 112–120. 29631001 10.1016/j.nlm.2018.04.003

[ejp70113-bib-0037] Mansour, A. R. , M. A. Farmer , M. N. Baliki , and A. V. Apkarian . 2014. “Chronic Pain: The Role of Learning and Brain Plasticity.” Restorative Neurology and Neuroscience 32: 129–139. 10.3233/RNN-139003. 23603439 PMC4922795

[ejp70113-bib-0038] Matthie, N. S. , N. A. Giordano , C. M. Jenerette , et al. 2022. “Use and Efficacy of Virtual, Augmented, or Mixed Reality Technology for Chronic Pain: A Systematic Review.” Pain Management 12, no. 7: 859–878. 10.2217/pmt-2022-0030. 36098065 PMC9517958

[ejp70113-bib-0039] Medina, S. , S. Clarke , and S. Hughes . 2024. Virtual Reality‐Based Analgesia: Towards a Novel Framework for the Biopsychosocial Management of Chronic Pain. Elsevier. 10.1016/j.bja.2024.06.005 38997839

[ejp70113-bib-0040] Medina, S. , and S. Hughes . 2024. “Immersion in Nature Attenuates the Development of Mechanical Secondary Hyperalgesia: A Role for Insulo‐Thalamic Effective Connectivity.” *bioRxiv*. 2024.2010.2011.617804. 10.1097/j.pain.0000000000003701 40717606

[ejp70113-bib-0041] Mehesz, E. , H. Karoui , P. H. Strutton , and S. W. Hughes . 2021. “Exposure to an Immersive Virtual Reality Environment Can Modulate Perceptual Correlates of Endogenous Analgesia and Central Sensitization in Healthy Volunteers.” Journal of Pain 22, no. 6: 707–714. 33465506 10.1016/j.jpain.2020.12.007

[ejp70113-bib-0042] Meijs, S. , F. R. Andreis , T. A. M. Janjua , T. Graven‐Nielsen , and W. Jensen . 2025. “High‐Frequency Electrical Stimulation Increases Cortical Excitability and Mechanical Sensitivity in a Chronic Large Animal Model.” Pain. 10.1097/j.pain.0000000000003354. 39133034

[ejp70113-bib-0043] Mulders, D. , B. Seymour , A. Mouraux , and F. Mancini . 2023. “Confidence of Probabilistic Predictions Modulates the Cortical Response to Pain.” Proceedings of the National Academy of Sciences 120, no. 4: e2212252120. 10.1073/pnas.2212252120. PMC9942789 36669115

[ejp70113-bib-0044] Nees, F. , and S. Becker . 2018. “Psychological Processes in Chronic Pain: Influences of Reward and Fear Learning as Key Mechanisms—Behavioral Evidence, Neural Circuits, and Maladaptive Changes.” Neuroscience 387: 72–84. 10.1016/j.neuroscience.2017.08.051. 28890049

[ejp70113-bib-0045] Nickel, M. M. , L. Tiemann , V. D. Hohn , et al. 2022. “Temporal–Spectral Signaling of Sensory Information and Expectations in the Cerebral Processing of Pain.” Proceedings of the National Academy of Sciences of the United States of America 119, no. 1: e2116616119. 10.1073/pnas.2116616119. 34983852 PMC8740684

[ejp70113-bib-0046] Palmisani, F. , S. Medina , and S. W. Hughes . 2024. A Novel VR‐Based Prediction Error Paradigm During Peripheral Sensitisation. OSF.

[ejp70113-bib-0047] Pavy, F. , J. Zaman , A. Von Leupoldt , and D. M. Torta . 2024. “Expectations Underlie the Effects of Unpredictable Pain: A Behavioral and Electroencephalogram Study.” Pain 165, no. 3: 596–607. 10.1097/j.pain.0000000000003046. 37703404

[ejp70113-bib-0048] Petrovic, P. , E. Kalso , K. M. Petersson , and M. Ingvar . 2002. “Placebo and Opioid Analgesia—Imaging a Shared Neuronal Network.” Science 295, no. 5560: 1737–1740. 10.1126/science.1067176. 11834781

[ejp70113-bib-0049] Ploghaus, A. , I. Tracey , S. Clare , J. S. Gati , J. N. P. Rawlins , and P. M. Matthews . 2000. “Learning About Pain: The Neural Substrate of the Prediction Error for Aversive Events.” Proceedings of the National Academy of Sciences 97, no. 16: 9281–9286. 10.1073/pnas.160266497. PMC16859 10908676

[ejp70113-bib-0050] Ramne, M. , and J. Sensinger . 2024. “A Computational Framework for Understanding the Impact of Prior Experiences on Pain Perception and Neuropathic Pain.” PLoS Computational Biology 20, no. 10: e1012097. 39480877 10.1371/journal.pcbi.1012097 PMC11556707

[ejp70113-bib-0051] Rowe, E. G. , N. Tsuchiya , and M. I. Garrido . 2020. “Detecting (Un)seen Change: The Neural Underpinnings of (Un)conscious Prediction Errors.” Frontiers in Systems Neuroscience 14: 541670. 10.3389/fnsys.2020.541670. 33262694 PMC7686547

[ejp70113-bib-0052] Roy, M. , D. Shohamy , N. Daw , M. Jepma , G. E. Wimmer , and T. D. Wager . 2014. “Representation of Aversive Prediction Errors in the Human Periaqueductal Gray.” Nature Neuroscience 17, no. 11: 1607–1612. 10.1038/nn.3832. 25282614 PMC4213247

[ejp70113-bib-0053] Ruscheweyh, R. , O. Wilder‐Smith , R. Drdla , X.‐G. Liu , and J. Sandkühler . 2011. “Long‐Term Potentiation in Spinal Nociceptive Pathways as a Novel Target for Pain Therapy.” Molecular Pain 7: 1744‐8069. 10.1186/1744-8069-7-20. PMC3078873 21443797

[ejp70113-bib-0054] Salagean, A. , J. Hadnett‐Hunter , D. J. Finnegan , A. A. D. Sousa , and M. J. Proulx . 2022. “A Virtual Reality Application of the Rubber Hand Illusion Induced by Ultrasonic Mid‐Air Haptic Stimulation.” ACM Transactions on Applied Perception 19, no. 1: 1–19. 10.1145/3487563.

[ejp70113-bib-0055] Schenk, L. A. , C. Sprenger , S. Onat , L. Colloca , and C. Büchel . 2017. “Suppression of Striatal Prediction Errors by the Prefrontal Cortex in Placebo Hypoalgesia.” Journal of Neuroscience 37, no. 40: 9715–9723. 10.1523/jneurosci.1101-17.2017. 28883019 PMC5628411

[ejp70113-bib-0056] Seymour, B. 2019. “Pain: A Precision Signal for Reinforcement Learning and Control.” Neuron 101, no. 6: 1029–1041. 10.1016/j.neuron.2019.01.055. 30897355

[ejp70113-bib-0057] Seymour, B. , and F. Mancini . 2020. “Hierarchical Models of Pain: Inference, Information‐Seeking, and Adaptive Control.” NeuroImage 222: 117212. 10.1016/j.neuroimage.2020.117212. 32739554

[ejp70113-bib-0058] Song, Y. , H. Kemprecos , J. Wang , and Z. Chen . 2019. “A Predictive Coding Model for Evoked and Spontaneous Pain Perception.” 2019 41st Annual International Conference of the IEEE Engineering in Medicine and Biology Society (EMBC), 23–27 July 2019. 10.1109/EMBC.2019.8857298 PMC8025043 31946512

[ejp70113-bib-0059] Song, Y. , M. Yao , H. Kemprecos , et al. 2021. “Predictive Coding Models for Pain Perception.” Journal of Computational Neuroscience 49, no. 2: 107–127. 10.1007/s10827-021-00780-x. 33595765 PMC8046732

[ejp70113-bib-0060] Strigo, I. A. , M. Kadlec , J. M. Mitchell , and A. N. Simmons . 2024. “Identification of Group Differences in Predictive Anticipatory Biasing of Pain During Uncertainty: Preparing for the Worst but Hoping for the Best.” Pain 165, no. 8: 1735–1747. https://journals.lww.com/pain/fulltext/2024/08000/identification_of_group_differences_in_predictive.10.aspx. 38501988 10.1097/j.pain.0000000000003207 PMC11247452

[ejp70113-bib-0061] Strube, A. , M. Rose , S. Fazeli , and C. Büchel . 2021. “The Temporal and Spectral Characteristics of Expectations and Prediction Errors in Pain and Thermoception.” eLife 10: e62809. 10.7554/eLife.62809. 33594976 PMC7924946

[ejp70113-bib-0062] Tabor, A. , and C. Burr . 2019. “Bayesian Learning Models of Pain: A Call to Action.” Current Opinion in Behavioral Sciences 26: 54–61. 10.1016/j.cobeha.2018.10.006.

[ejp70113-bib-0063] Thomaidou, M. A. , D. S. Veldhuijzen , A. Meulders , and A. W. M. Evers . 2021. “An Experimental Investigation Into the Mediating Role of Pain‐Related Fear in Boosting Nocebo Hyperalgesia.” Pain 162, no. 1: 287–299. https://journals.lww.com/pain/fulltext/2021/01000/an_experimental_investigation_into_the_mediating.25.aspx. 32910630 10.1097/j.pain.0000000000002017 PMC7737877

[ejp70113-bib-0064] Timmers, I. , C. W. E. M. Quaedflieg , C. Hsu , L. C. Heathcote , C. R. Rovnaghi , and L. E. Simons . 2019. “The Interaction Between Stress and Chronic Pain Through the Lens of Threat Learning.” Neuroscience & Biobehavioral Reviews 107: 641–655. 10.1016/j.neubiorev.2019.10.007. 31622630 PMC6914269

[ejp70113-bib-0065] Torta, D. M. , M. De Laurentis , K. N. Eichin , A. von Leupoldt , E. N. van den Broeke , and J. W. S. Vlaeyen . 2020. “A Highly Cognitive Demanding Working Memory Task May Prevent the Development of Nociceptive Hypersensitivity.” Pain 161, no. 7: 1459–1469. 10.1097/j.pain.0000000000001841. 32102023

[ejp70113-bib-0066] Torta, D. M. , E. Meyers , K. Polleunis , S. De Wolf , A. Meulders , and E. N. van den Broeke . 2023. “The Effect of Observing High or Low Pain on the Development of Central Sensitization.” Journal of Pain 24, no. 1: 167–177. 10.1016/j.jpain.2022.09.009. 36162789

[ejp70113-bib-0067] Tracey, I. 2010. “Getting the Pain You Expect: Mechanisms of Placebo, Nocebo and Reappraisal Effects in Humans.” Nature Medicine 16, no. 11: 1277–1283. 10.1038/nm.2229. 20948533

[ejp70113-bib-0068] Traxler, J. , D. M. Torta , A. von Leupoldt , and J. W. S. Vlaeyen . 2022. “Error Processing and Pain: A New Perspective.” Journal of Pain 23, no. 11: 1811–1822. 10.1016/j.jpain.2022.05.005. 35643271

[ejp70113-bib-0069] Tsai, H.‐Y. , K. Lapanan , Y.‐H. Lin , et al. 2024. “Integration of Prior Expectations and Suppression of Prediction Errors During Expectancy‐Induced Pain Modulation: The Influence of Anxiety and Pleasantness.” Journal of Neuroscience 44, no. 17: e1627‐232024. 10.1523/jneurosci.1627-23.2024. PMC11044194 38453467

[ejp70113-bib-0070] van Amerongen, G. , M. W. de Boer , G. J. Groeneveld , and J. L. Hay . 2016. “A Literature Review on the Pharmacological Sensitivity of Human Evoked Hyperalgesia Pain Models.” British Journal of Clinical Pharmacology 82, no. 4: 903–922. 27203797 10.1111/bcp.13018 PMC5276025

[ejp70113-bib-0071] van den Broeke, E. N. , N. Geene , C. M. van Rijn , O. H. G. Wilder‐Smith , and J. Oosterman . 2014. “Negative Expectations Facilitate Mechanical Hyperalgesia After High‐Frequency Electrical Stimulation of Human Skin.” European Journal of Pain 18, no. 1: 86–91. 10.1002/j.1532-2149.2013.00342.x. 23754275

[ejp70113-bib-0072] Van den Broeke, E. N. , J. Lambert , G. Huang , and A. Mouraux . 2016. “Central Sensitization of Mechanical Nociceptive Pathways Is Associated With a Long‐Lasting Increase of Pinprick‐Evoked Brain Potentials.” Frontiers in Human Neuroscience 10: 531. 27812331 10.3389/fnhum.2016.00531 PMC5071355

[ejp70113-bib-0073] van den Broeke, E. N. , and A. Mouraux . 2014. “High‐Frequency Electrical Stimulation of the Human Skin Induces Heterotopical Mechanical Hyperalgesia, Heat Hyperalgesia, and Enhanced Responses to Nonnociceptive Vibrotactile Input.” Journal of Neurophysiology 111, no. 8: 1564–1573. 10.1152/jn.00651.2013. 24453277

[ejp70113-bib-0074] van den Broeke, E. N. , C. H. van Heck , C. M. van Rijn , and O. H. Wilder‐Smith . 2011. “Neural Correlates of Heterotopic Facilitation Induced After High Frequency Electrical Stimulation of Nociceptive Pathways.” Molecular Pain 7: 1744‐8069‐1747‐1728. 10.1186/1744-8069-7-28. PMC3108312 21507241

[ejp70113-bib-0075] Velasco, E. , M. Flores‐Cortés , J. Guerra‐Armas , et al. 2024. “Is Chronic Pain Caused by Central Sensitization? A Review and Critical Point of View.” Neuroscience & Biobehavioral Reviews 167: 105886. 10.1016/j.neubiorev.2024.105886. 39278607

[ejp70113-bib-0076] Vlaeyen, J. W. S. 2015. “Learning to Predict and Control Harmful Events: Chronic Pain and Conditioning.” Pain 156: S86–S93. https://journals.lww.com/pain/fulltext/2015/04001/learning_to_predict_and_control_harmful_events_.12.aspx. 25789440 10.1097/j.pain.0000000000000107

[ejp70113-bib-0077] Vo, L. , and P. D. Drummond . 2013. “High Frequency Electrical Stimulation Concurrently Induces Central Sensitization and Ipsilateral Inhibitory Pain Modulation.” European Journal of Pain 17, no. 3: 357–368. 10.1002/j.1532-2149.2012.00208.x. 22893547

[ejp70113-bib-0078] Vo, L. , and P. D. Drummond . 2016. “Involvement of α2‐Adrenoceptors in Inhibitory and Facilitatory Pain Modulation Processes.” European Journal of Pain 20, no. 3: 386–398. 10.1002/ejp.736. 26032281

[ejp70113-bib-0079] Volcheck, M. M. , S. M. Graham , K. C. Fleming , A. B. Mohabbat , and C. A. Luedtke . 2023. “Central Sensitization, Chronic Pain, and Other Symptoms: Better Understanding, Better Management.” Cleveland Clinic Journal of Medicine 90, no. 4: 245–254. 10.3949/ccjm.90a.22019. 37011956

[ejp70113-bib-0080] Vollert, J. , N. Attal , R. Baron , et al. 2016. “Quantitative Sensory Testing Using DFNS Protocol in Europe: An Evaluation of Heterogeneity Across Multiple Centers in Patients With Peripheral Neuropathic Pain and Healthy Subjects.” Pain 157, no. 3: 750–758. 26630440 10.1097/j.pain.0000000000000433

[ejp70113-bib-0081] Vollert, J. , W. Magerl , R. Baron , et al. 2018. “Pathophysiological Mechanisms of Neuropathic Pain: Comparison of Sensory Phenotypes in Patients and Human Surrogate Pain Models.” Pain 159, no. 6: 1090–1102. 10.1097/j.pain.0000000000001190. 29494416

[ejp70113-bib-0082] Wager, T. D. , L. Y. Atlas , L. A. Leotti , and J. K. Rilling . 2011. “Predicting Individual Differences in Placebo Analgesia: Contributions of Brain Activity During Anticipation and Pain Experience.” Journal of Neuroscience 31, no. 2: 439–452. 10.1523/jneurosci.3420-10.2011. 21228154 PMC3735131

[ejp70113-bib-0083] Wager, T. D. , D. J. Scott , and J.‐K. Zubieta . 2007. “Placebo Effects on Human μ‐Opioid Activity During Pain.” Proceedings of the National Academy of Sciences 104, no. 26: 11056–11061. 10.1073/pnas.0702413104. PMC1894566 17578917

[ejp70113-bib-0084] Woolf, C. J. 2011. “Central Sensitization: Implications for the Diagnosis and Treatment of Pain.” Pain 152, no. 3S: S2–S15. 10.1016/j.pain.2010.09.030. 20961685 PMC3268359

[ejp70113-bib-0085] Yakunchikov, D. Y. , C. J. Olechowski , M. K. Simmonds , et al. 2016. “The Effect of Social Observational Learning, Empathy and Catastrophizing in Chronic Pain Patients During Acute Pain Induction.” Pain Medicine 18, no. 5: 871–878. 10.1093/pm/pnw186. 27561307

[ejp70113-bib-0086] Zhang, L. , E. A. R. Losin , Y. K. Ashar , L. Koban , and T. D. Wager . 2021. “Gender Biases in Estimation of Others' Pain.” Journal of Pain 22, no. 9: 1048–1059. 10.1016/j.jpain.2021.03.001. 33684539 PMC8827218

